# Letter to the Editor of *Metabolites*

**DOI:** 10.3390/metabo10050216

**Published:** 2020-05-25

**Authors:** Maureen R. Hanson, Arnaud Germain

**Affiliations:** Department of Molecular Biology and Genetics, Cornell University, Biotechnology Building, Ithaca, NY 14853, USA; ag297@cornell.edu

**Keywords:** ME/CFS, prevalence, worldwide, review

Dear Editor,

A number in our recently published study in your *Metabolites* journal, Germain et al. (2020), entitled “Comprehensive Circulatory Metabolomics in ME/CFS Reveals Disrupted Metabolism of Acyl Lipids and Steroids” [1] has drawn a significant amount of attention on social media. We stated that the worldwide prevalence rate of ME/CFS is likely “over 65 million patients.” Unfortunately, to support this figure, we cited only a paper by Valdez et al. (2018), titled “Estimating prevalence, demographics, and costs of ME/CFS using large scale medical claims data and machine learning” [2], in which all the data was gathered in the USA. According to their carefully selected dataset based on U.S. medical records, the frequency of U.S. diagnosis ranges from 0.52% to 1.04%, giving a take-home prevalence rate in their analysis of 0.86%. With a U.S. population of roughly 330 million Americans, the estimated patient population in the U.S. is 2.8 million, ranging from 1.7 to 3.4 million [2].

When we extrapolated from their study, we selected the middle prevalence rate of 0.86% for a world population of 7.8 billion humans, giving a total of 67 million patients worldwide. The actual worldwide prevalence of ME/CFS is difficult to determine because although there are a multitude of reports concerning the issue, the lack of an easily applied objective diagnostic test means that diagnosis must be done through symptom constellation. Because different studies have applied a variety of diagnostic criteria, there is substantial variation in prevalence estimates. Nevertheless, a close look at these reports indicates that it is not unreasonable to use the 0.86% prevalence rate worldwide.

A review by Son (2012) [3] curated 34 manuscripts focusing on ME/CFS, totaling almost 450,000 subjects from 11 countries (Australia, Brazil, China, Netherlands, Iceland, Israel, Japan, South Korea, Sweden, United Kingdom, and USA) published between 1990 and 2011. When restricting the prevalence rate to the 24 papers using cohorts from the general population, he obtained an average of 1.2%. The Son group has updated the 2012 study this year with an impressive follow-up evaluation [4]. Using 45 articles, their meta-analysis gave an estimate of 0.65%, but with the 1994 Fukuda criteria, yielded a prevalence of 0.89%, with a 1.5 to 2-fold predominance of women. This figure is remarkably similar to that found by Valdez et al. (2018), though an important caveat is the high heterogeneity in estimated rates.

A number of the prevalence estimates in prior studies are likely too low due to a variety of factors. For example, undiagnosed individuals would not be able to identify their condition if they provided blood to a biobank, and ones who are extremely ill are not as likely to provide samples. Such issues could have contributed to the prevalence of only 0.45% in an analysis of the 500,000-member United Kingdom (UK) Biobank cohort [5]. The disbelief in the disease as a physical illness by some medical professionals, ignorance of its existence in children, as well as reduced access to medical care by individuals of lower socioeconomic status, may contribute to inappropriately low rates of diagnosis, resulting in inaccurate estimations of prevalence [6].

A particularly notable paper is the 2020 analysis of pediatric prevalence in a Chicago population, which through careful analysis found a rate of 0.75%, even though less than 5% of the subjects had been previously diagnosed with the illness. While most studies of the biological basis of ME/CFS have mainly had access to Caucasian subjects, in the pediatric study, African Americans and Latinx had a higher percentage of illness than Caucasians [7].

To conclude, we think it is crucial to recognize both the studies done so far and their limitations. When a simple diagnostic test is available for ME/CFS, the true incidence of the disease worldwide will be able to be more accurately determined. However, a figure of 65 million, extrapolated not only from medical record mining in the US, but also from studies from other countries, is actually a relatively conservative estimate. If the commonly reported figure of approximately 1% is utilized, then there are 78 million sufferers, and if 1.2%, there are about 100 million. Prevalence numbers must remain estimates at this time. Nevertheless, as written by Valdez et al. (2018), ME/CFS “is not a rare disease, but in fact a relatively common one”.

## References

[1] Germain A., Barupal D.K., Levine S.M., Hanson M.R. (2020). Comprehensive circulatory metabolomics in ME/CFS reveals disrupted metabolism of acyl lipids and steroids. Metabolites.

[2] Valdez A.R., Hancock E.E., Adebayo S., Kiernicki D.J., Proskauer D., Attewell J.R., Bateman L., DeMaria A., Lapp C.W., Rowe P.C. (2018). Estimating prevalence, demographics, and costs of ME/CFS using large scale medical claims data and machine learning. Front. Pediatr..

[3] Son C. (2012). Review of the prevalence of chronic fatigue worldwide. J. Korean Orient. Med..

[4] Lim E.J., Ahn Y.C., Jang E.S., Lee S.W., Lee S.H., Son C.G. (2020). Systematic review and meta-analysis of the prevalence of chronic fatigue syndrome/myalgic encephalomyelitis (CFS/ME). J. Transl. Med..

[5] Canela-Xandri O., Rawlik K., Tenesa A. (2018). An atlas of genetic associations in UK Biobank. Nat. Genet..

[6] Collin S.M., Bakken I.J., Nazareth I., Crawley E., White P.D. (2017). Trends in the incidence of chronic fatigue syndrome and fibromyalgia in the UK, 2001–2013: A Clinical Practice Research Datalink study. J. R. Soc. Med..

[7] Jason L.A., Katz B.Z., Sunnquist M., Torres C., Cotler J., Bhatia S. (2020). The prevalence of pediatric myalgic encephalomyelitis/chronic fatigue syndrome in a community-based sample. Child Youth Care Forum.

